# Audience effects in a group‐living bird: How contact call rate is affected by vegetation and group size and composition

**DOI:** 10.1002/ece3.9909

**Published:** 2023-03-24

**Authors:** Estelle Meaux, Chao He, Xiaolei Zeng, Ruchuan He, Aiwu Jiang, Eben Goodale

**Affiliations:** ^1^ Guangxi Key Laboratory of Forest Ecology and Conservation College of Forestry, Guangxi University Nanning Guangxi China; ^2^ Department of Health and Environmental Science Xi'an Jiaotong‐Liverpool University Suzhou Jiangsu China

**Keywords:** acoustic signal, animal sociality, calling rate, passerine species, social stimulation, vocal communication

## Abstract

Contact calling is a ubiquitous behavior of group‐living animals. Yet in birds, beyond a general connection with group cohesion, its precise function is not well‐understood, nor is it clear what stimulates changes in contact call rate. In an aviary experiment, we asked whether Swinhoe's White‐eyes, *Zosterops simplex*, would regulate their own production of contact calls to maintain a specific rate at the group level. Specifically, we hypothesized that the sudden cessation of the group‐level call rate could indicate an immediate predation threat, and we predicted that birds in smaller groups would call more to maintain a high call rate. We also investigated the effects of environmental characteristics, such as vegetation density, and social stimuli, such as the presence of certain individuals, on the rate of three different contact call types. To calculate mean individual‐level rates, we measured the group‐level rate and divided it by the number of birds in the aviary. We found that the individual‐level rate of the most common call types increased with a greater group size, the opposite pattern to what would be expected if birds were maintaining a specific group‐level rate. Vegetation density did not affect any call rate. However, individual‐level rates of all call types decreased when birds were in subgroups with individuals of differing dominance status, and the rate of some call types increased when birds were with affiliated individuals. Our results do not support the hypothesis that contact calls are related to habitat structure or immediate predation risk. Rather, they appear to have a social function, used for communication within or between groups depending on the call type. Increases in call rates could recruit affiliated individuals, whereas subordinates could withhold calls so that dominants are unable to locate them, leading to fluctuations in contact calling in different social contexts.

## Graphical abstract

The influence of group size on the contact call rate depends on the type of call. Contact call rate was not related to vegetation density. Call rates changed with the presence of group members of certain rank or affiliation.

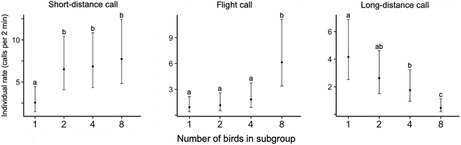

## 1 INTRODUCTION

Group‐living animals face a fundamental problem in maintaining group cohesion, needing to stay aware of the locations of other group members and the direction of their movement (Boinski & Garber, 2000). For animals that must move through thick vegetation or that must spend much visual attention on foraging, an important mechanism for maintaining cohesion are vocalizations, and in particular “contact calls” that are produced in a repetitive manner by most group members (Fichtel & Manser, 2010; Kondo & Watanabe, 2009; Marler, 2004; Snijders & Naguib, 2017). We will hereafter use the term “contact call” to refer broadly to any vocalization related to group movement, spacing or cohesion, following Meaux, Peabotuwage, et al. (2021). Much of the research on contact calls has focused on their acoustic characteristics, and it has been shown that they can allow the recognition of individuals (reviewed by Rendall & Owren, 2002), or can be an indicator of group membership, through convergence of calls' acoustic properties (reviewed by Sewall, 2015). Surprisingly, few studies have looked at one of the most distinctive properties of these repeatedly produced calls: their rate of production. Contact call rate has been investigated primarily in mammal species (Boinski, 1991; Engesser & Manser, 2022; Ey et al., 2009; Mausbach et al., 2017; Sperber et al., 2017), with less research on birds (but see Radford & Ridley, 2008; Striedter et al., 2003). In species in which group size is dynamic, for example, in fission–fusion societies, the group‐level rate of contact calling could be a source of information about how many individuals are present. For more stable groups, however, group‐level contact call rate is determined by individuals modulating their own call rate away from the baseline level. Here, we concentrate on what kind of stimuli might elicit such changes in individual‐level and group‐level contact call rates in relatively stable groups.

Although it is clear that contact calls are somehow related to group cohesion, their exact function can vary between species and has often not been precisely understood. In some social systems, contact calls appear to regulate spacing between individuals to avoid intraspecific competition (Bolt & Tennenhouse, 2017; Mitani & Nishida, 1993; Radford & Ridley, 2008). In other systems, contact call rate is more closely linked to group movement and can increase when a group is about to depart (Boinski, 1991; Sperber et al., 2017). Contact calls can also initiate movements toward a “hot spot” where the rate is highest, as observed in suricates, *Suricata suricatta* (Gall & Manser, 2017), suggesting a form of recruitment. The recruitment function of contact calls has also been suggested by studies focusing on birds, such as in house sparrows, *Passer domesticus* (Elgar, 1986), in pied babblers, *Turdoides bicolor* (Engesser et al., 2018), or in the Carolina chickadee, *Poecile carolinensis*, and other parid species (Freeberg & Mahurin, 2013; Hillemann et al., 2019). At the same time, contact calls can have social functions regulating the relationships between individuals, perhaps linked to their distinctiveness at the individual level, which would potentially allow birds to be aware not only of the location of other individuals, but also of their identity (see e.g., in meerkats, Reber et al., 2013). For example, some primates have been observed to increase contact calling rate when associating with affiliated individuals, which has been described as “grooming‐at‐distance” (Arlet et al., 2015; Kulahci et al., 2015).

Given these varied functions of contact calls, the stimuli that elicit changes in individuals' call rates can also be diverse. For functions involving movement, the stimulus may be the location or vocalization of other group members. For social functions, call production may be triggered by the location or vocalization of particular individuals, depending on their social relationship with the signaller. However, stimuli could also be related to environmental context. For example, if contact calls function to give information about the location of the caller, such as when they regulate spacing between flock members, then we might expect calling rates to be higher in denser vegetation in which visual information about location is obstructed (Ey et al., 2009; Hedwig et al., 2015; Hollén et al., 2011; Kern & Radford, 2013; Krama et al., 2008).

Another potential function of contact calling that requires further study is related to predation risk. The continual nature of contact calling could function as a signal of safety, indicating immediate predation risk (i.e., the presence of a predator) when calling suddenly ceases. The general idea of silence serving as information about immediate predation risk in noisy groups of animals was termed “adaptive silence” by Curio (1978). This behavior has been observed in various vertebrate and invertebrate taxa (primates: Miranda et al., 2006; rodents: Pereira et al., 2012; amphibians: Dapper et al., 2011; fish: Luczkovich et al., 2000; insects: Spangler, 1984). To our knowledge, this behavior has not been studied in relation to the contact calls of birds, although a recent study by Lilly et al. (2019) showed that the gray squirrel, *Sciurus carolinensis*, could interpret the cessation of birds “chatter” as a signal of danger. A related but more specific phenomenon can be found in sentinel animals, in which there are certain guarding individuals making repetitive vocalizations (“the watchman's song”) that indicate their vigilance status. This has been studied in highly social mammals such as dwarf mongooses, *Helogale parvula* (Kern & Radford, 2013) and suricates (Manser, 1999), as well as in a few avian species (Bell et al., 2009; Wingelmaier et al., 2007). In addition, some social birds have specific contact call types used in contexts of high predation risk (Sieving et al., 2010). Importantly, if the cessation of contact calls is used as an indication of immediate risk, there may be a need to maintain an optimal group‐level rate of contact calling, as too low a rate might make a shift to silence imperceptible. If that is the case, we could expect individuals to adjust their own call production depending on the group size, and specifically to increase their calling rate when fewer individuals are present.

In this study, we worked with a highly gregarious passerine species, Swinhoe's White‐eye, *Zosterops simplex*. Flocks of white‐eyes can be quite stable, formed by the same individuals and found in the same place day after day (Kikkawa, 1961; Meaux, Peabotuwage, et al., 2021), and produce nearly continual contact calls, which makes this species a good model to study the factors modulating contact call rate. A first objective in this study was to determine whether white‐eyes would regulate their individual calling rates to maintain a group‐level rate. Specifically, we hypothesized that in smaller groups, individuals would make contact calls at a higher rate, so that the rate would remain high, and thus the cessation of calling would be a perceptible indication of predation risk. We manipulated group size in an aviary experiment, measuring the group‐level calling frequency of the birds, and then dividing it by the number of individuals present to calculate a mean individual‐level calling rate (hereafter, we refer to this as simply as “individual‐level” rate). This study can thus be considered as a kind of “audience experiment” that tests whether the presence of certain receivers influence the production of communicatory signals (see reviews by Coppinger et al. (2017); Townsend et al. (2017); Zuberbühler (2008)). A second objective was to understand what factors would cause changes in individual‐level contact call rates. We investigated whether white‐eyes would change their contact call rate due to an environmental characteristic, the vegetation density, or to a social stimulus, the presence of particular individuals in the composition of the group. Previous fieldwork has shown in this species that the group‐level contact call rate is not strongly influenced by the vegetation where the birds are located (Meaux, Peabotuwage, et al., 2021); however, we wanted to re‐examine this finding under more controlled conditions. Since birds' rate of vocalizations are often influenced by their social setting (Coppinger et al., 2018; Elie et al., 2011; Fernandez et al., 2017; Freeberg & Harvey, 2008), we expected that the presence of affiliated individuals together in a group would increase the individual‐level call rate. We also noticed that white‐eyes have a strong dominance hierarchy, notably when competing for access to a feeder, and predicted that calling rate would be lower when birds of opposite dominance status were present together, specifically due to subordinates withholding information about their location (Wiley, 1983). Finally, like other contact call systems (Ey & Fischer, 2011; Krama et al., 2008; Krams, 2001), white‐eyes use distinct contact call types, including soft “short‐distance” and louder “long‐distance” calls, as well as a “flight call,” which is only produced by a flying individual (Meaux, He, et al., 2021; Robertson, 1996). We therefore expected different contact call types would show different relationships with group size, predicting at the individual‐level: (a) an increase in short‐distance calls in larger groups when there are more individuals nearby the signaller; (b) a high production of long‐distance calls at reduced group sizes when individuals may signal that they have become isolated from the group; (c) an increase in flight calls in larger groups within which individuals may fly more frequently due to higher competition and/or aggression.

## 2 METHODS

### 2.1 Ethical note

The study was performed in accordance with the Guide to the Use of Animals in Research of the Animal Behaviour Society and with relevant institutional and national guidelines for the care and use of laboratory animals. The experimental protocol and procedures employed were ethically reviewed and approved by the Animal Experimental Ethical Inspection of Guangxi University (GXU2018‐038). The catching permit was issued by the Agricultural, Forestry, and Water Conservancy Bureau of the Xixiangtang District, Nanning City, P.R. China.

### 2.2 Subjects and captive conditions

Swinhoe's White‐eye, *Zosterops simplex* (formerly considered a subspecies of *Zosterops japonicus*, see Lim et al., 2019), is a highly adaptable group‐living species that can live in human‐disturbed areas, and is commonly kept as a caged bird in mainland China. Group size is variable and can range from four to 20 or more individuals; for example, in a previous study in Yunnan Province, P.R. China, groups averaged 9.25 ± [SD] 6.61 individuals (*N* = 810; Meaux, Peabotuwage, et al., 2021). Since we conducted experiments in captivity with limited space, we selected to test groups of eight birds. We caught birds using a traditional trapping technique (see Appendix S1 for details on the capture methods). With the help of local people familiar with the technique, we captured the flocks in Guangxi University campus and surrounding green spaces of Xixiangtang District, Nanning, P.R. China. Because multiple traps were spread in a small area, it is likely that the captured birds were part of the same natural social group. Between May 2019 and June 2020, we captured 10 white‐eye flocks, one group at a time. Six groups had eight birds and four groups had seven individuals (three groups were caught with only seven birds; and one group of eight had a bird that died during the pre‐experimental phase).

After capture, the subjects were placed in captivity in a 7 m long x 3 m wide x 2.5 m high open‐air aviary that was adjacent to a work room with an observation window (see Appendix S1 for details on aviary conditions). Once a group was placed in captivity, it was observed for 5–8 days during a pre‐experimental, habituation period. After this, the group was subjected to the group size and vegetation experiments, as well as other simultaneous experiments discussed elsewhere (Meaux, He, et al., 2021), for on average seven additional days, before being released at the capture site. Sometimes wild white‐eyes were present in the area, but because of the lack of trees near the aviary, they did not get within 10 m of it. During all experiments, we observed and noted whether wild white‐eyes were present and vocalizing within ~100 m of the aviary. All captive individuals were color‐banded for visual identification upon capture and measured. They were later blood sampled before release to allow sexual identification for this monomorphic species. Since the rings were left on the birds' legs after release, this ensures that we were not re‐catching the same individuals when capturing another flock.

### 2.3 Pre‐experimental protocols

To characterize the social dynamics of the group, we made observations on the agonistic and affiliative behavior of the birds during the pre‐experimental phase (see Table S1 in Appendix S1). On the last 2 days of this phase, we also recorded their vocalizations twice a day (morning and afternoon; see below for methods of recording). We later considered these 120‐s recordings as representative of birds in a full group size and without any vegetation in the aviary beyond perching structures (see Section 2.6: Statistical analyses). During these observations, the number of active birds was noted (a bird was considered inactive when no change of location occurred for more than 60 s).

A social hierarchy test was conducted once a day before feeding to measure the social ranking of the subjects. For this purpose, all the food was removed from the aviary, and then a large piece of fruit was placed on a raised stool in the middle of the aviary. Agonistic behaviors were then recorded by video camera (JVC EverioR GZ‐R465, situated on a tripod 2–3 m away) for 10 min before the test ended. This sampling effort was enough to record the minimum number of interactions necessary to determine reliable social ranking, that is, 80–160 interactions for a group of eight individuals (Sánchez‐Tójar et al., 2018). Behaviors were then coded using BORIS version 7.4.15 (Friard & Gamba, 2016) to analyze the dominance encounters and build a matrix of dominance relationships. Finally, the normalized David's score was calculated to infer the hierarchy within each group (De Vries et al., 2006).

To measure affiliation among subjects, we observed each bird (chosen in a random order) for a 2‐min focal sample (Altmann, 1974), twice a day. During these observations, the identity of any near neighbor (a bird within three body lengths from the subject) was recorded. We also measured the duration of three kinds of mutually exclusive interactions of the focal and neighboring birds: (a) allopreening; (b) staying in other kinds of physical contact (e.g., sleeping while touching each other); and (c) interactions without physical contact. These data were used to build a matrix of affiliation scores for each pair of birds as the time spent with each other (in s). All agonistic interactions observed during this focal sampling were added into the dominance data.

### 2.4 Acoustic analyses

The subjects' vocalizations were recorded from the work room with a directional shotgun microphone (Sennheiser ME66) and recorded onto a digital recorder (Marantz PMD670) with 48 kHz, 16‐bit sampling. All audio samples were of 120 s and taken with the exact same volume settings on the recorder. At the beginning of the experiments, we determined that we could detect all the calls produced by the captive birds (see Appendix S1 for more details). The maximum distance between a subject and the microphone was 7 m (the length of the aviary).

**FIGURE 1:**
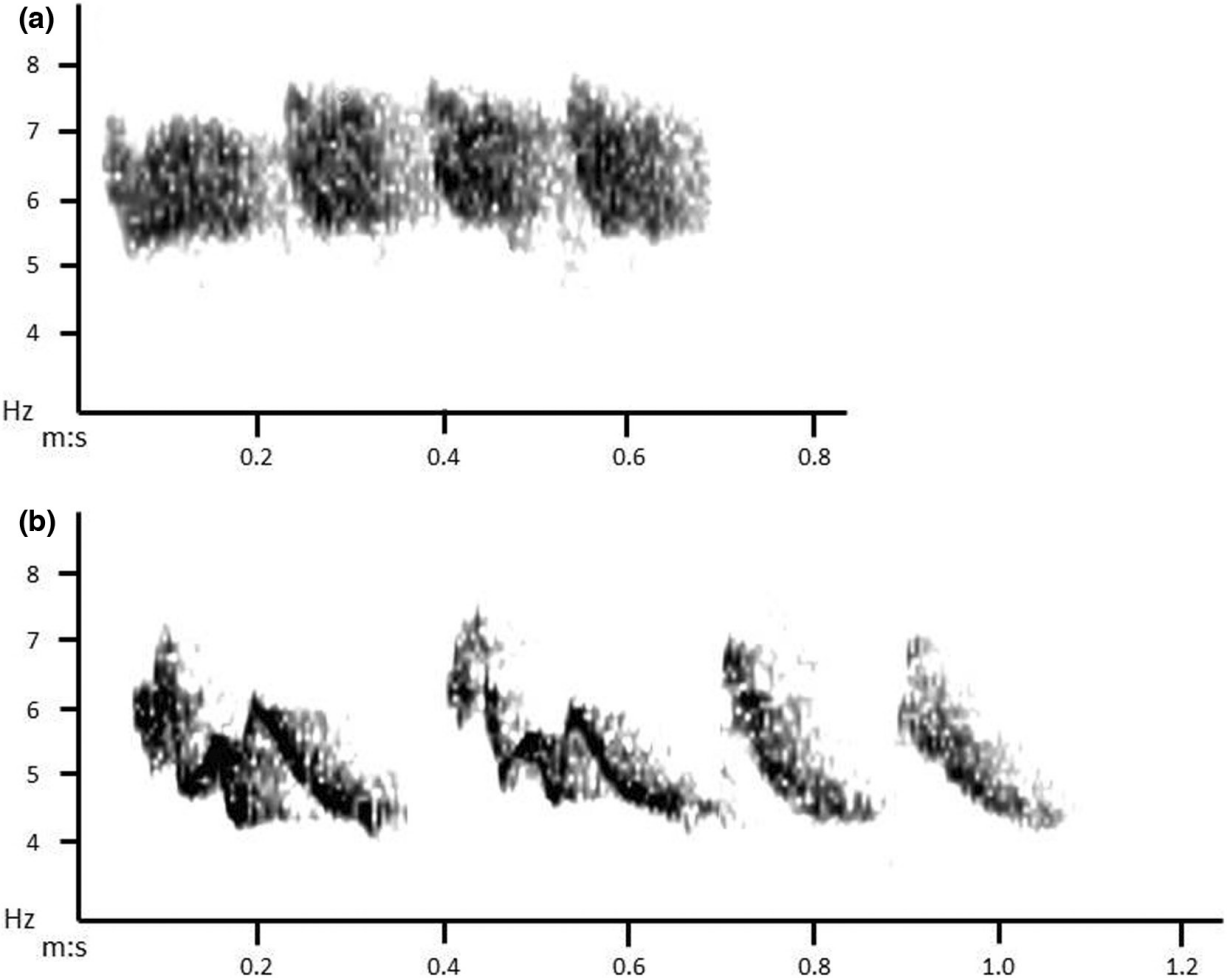
Spectrograms of contact calls of Swinhoe's White‐eye. Spectrograms were made using Raven Pro 1.5 with 512 FFT and a Hann Window. (a) Example of flight calls produced in a series of four notes; (b) Example of two long‐distance calls followed by two short‐distance calls.

We manually categorized contact calls into three types: a short‐distance call, a long‐distance call, and a flight call (Figure 1; see Results for their acoustic properties). To understand how discrete these call types were, we measured acoustic properties of 30 notes for each call type (three notes per white‐eye group, taken randomly from the sound files recorded during the pre‐experimental phase), using Raven Pro version 1.5 (Cornell Laboratory of Ornithology). We made five measurements of the notes: duration of the note, peak frequency of the beginning of the note, frequency modulation over the note, frequency bandwidth in the middle of the note, and peak amplitude of the note (see Appendix S1 for a detailed description of these measurements). In counting contact calls using Raven, we included all notes with a peak amplitude at least 10 dB greater than background noise (using a spectrum section of the note; a technique modified after Podos, 2001). Calls of wild white‐eyes were distinguished on the spectrogram because of their distinct reduced amplitude (see Section 3: Results) and excluded from the count of calls produced by the captive birds. The two observers classifying the calls (EM and CH) previously trained together to reach a level of accuracy >95%.

Finally, we were interested to know about individual differences in the acoustic properties of contact calls. For this, using Raven Pro, we analyzed the five recordings made in the subgroup of one individual, when there was a solitary bird in the aviary (see below for the experimental procedure). When birds were alone, they were not moving much and the flight calls were rare, so we investigated long‐distance and short‐distance calls only. For both call types, we randomly selected up to 10 notes per individual, trying to sample equally from the five different recordings when possible. The birds tended to be very silent, and some individuals were represented by less than 10 notes; birds with less than three notes during the 1‐h trial were excluded from the analysis. Therefore, the number of individuals analyzed was small (10 individuals for long‐distance calls and seven for short‐distance calls). We made measurements of the notes similar to the above, but not including amplitude, as it was not relevant to the question of whether contact calls are individually distinctive (for more details, see Appendix S1).

### 2.5 Experimental procedures

#### 2.5.1 Vegetation effect experiment

For this experiment, we temporarily introduced plastic vegetation imitating bamboo stems of ~2.5 m high (i.e., the height of the aviary) for the 1.5‐h duration trial. There were two treatments representing low‐ and high‐density vegetation. Low‐density vegetation was represented by one line of 17 stems of the fake bamboo (in the center of the aviary), and the high‐density vegetation treatment consisted of three such lines. These levels of vegetation density were such that a bird on the other side of the bamboo could still be visible at low density, but not at high density (as measured by human eyes). We used artificial bamboo because white‐eyes generally avoid perching in bamboo (unlike other vegetation we tried), so that its presence was purely a form of visual obstruction. Group members continued to forage throughout the aviary, on either side of the bamboo, so that in the high‐density treatment birds were subjected to considerable visual obstruction between them and other group members.

In these experiments, all group members were present. Recordings of the group started 30 min after setting up the vegetation, and a total of five recordings (each of 120 s) were made per trial, separated by 10 min from one another. During the experimental phase, a white‐eye group was tested in three trials for each density level, with the order of treatments assigned randomly, and with a maximum of two trials per day (each separated by at least 1 h). As in the pre‐experimental observations, the number of active birds was counted for each 120‐s recording, and we also noted any detection of wild birds' vocalizations.

#### 2.5.2 Group effect experiment

The experimental procedure of the group effect experiment was almost identical to the vegetation density experiment, except that instead of introducing artificial vegetation, we excluded some birds from the aviary. Specifically, we randomly selected and then excluded individuals from the main aviary in order to get subgroups of one, two, and four birds. Butterfly nets were used to capture nonfocal individuals, who were then temporally housed in a room where they could not be heard. Recording of the remaining individual(s) started 30 min after excluding the nonfocal subjects, and a trial consisted of making a total of five recordings of 120 s, each again separated by 10 min. In order to capture birds only once per day, and thereby reduce the birds' stress, we always started with the trial with group size of one, leaving only one bird in the aviary. After this first trial was completed, we released one of the other birds into the aviary, and waited for 30 min before recording the trial with group size of two. After all recordings with group size of two were made, we added two more birds, waited 30 min, and then conducted the trial with a group size of four. Similar to the vegetation density experiment, during the experimental phase, we conducted three 1.5‐h trials of each subgroup size for each group, so the procedure detailed above was repeated on three, usually consecutive, days. For each such trial, however, the subgroup was composed of different, randomly chosen individuals.

### 2.6 Statistical analyses

All tests were performed in R (version 3.6.1), with the alpha level set at 0.05.

### 2.7 Acoustic comparison of different kinds of contact calls

To see how repeatable our categorization of contact calls was, we conducted a linear discriminant analysis (LDA), with the type of contact call as response variable (short‐distance, long‐distance, and flight call) and the five acoustic measurements as fixed effects. The LDA was computed using the function “lda” in package “MASS” (Venables & Ripley, 2002).

### 2.8 Individual differences in acoustic properties

To reduce the number of variables (the six acoustic measurements), we conducted principal components analysis (PCA), using the “rda” function in package “vegan” (Oksanen et al., 2020). This analysis was followed by an ANOVA to investigate whether the identity of the individual explained the score of the recording on the first axis. This procedure was conducted separately for long‐distance calls and short‐distance calls.

### 2.9 Modeling of contact call rate

To understand the factors that influenced contact call rate, we performed generalized linear mixed models (GLMM), which allowed us to incorporate different kinds of replication as random factors, using the package “glmmTMB” (Brooks et al., 2017). For each experiment, we ran three separate models for the number of short‐distance, long‐distance, and flight calls produced during the 120‐s audio recordings. Models showed overdispersion, so we used negative binomial distributions. All variables showed a variance inflation factor below our preselected cutoff of three, demonstrating that multicollinearity was not problematic (Zuur et al., 2010). Multiple comparisons between subgroup sizes were conducted with Tukey's HSD adjustments made with the package “emmeans” (Lenth et al., 2018). We evaluated model fit by computing the marginal and conditional *R* ^*2*^ separately (Nakagawa & Schielzeth, 2013).

### 2.10 Vegetation effect experiment

For these experiments, the whole white‐eye group was present, so the group‐level call rate was the response variable. To make the number of contact calls in groups with seven individuals comparable to the other groups, we multiplied by 8/7. To incorporate the data when there was not any artificial vegetation during the pre‐experimental phase, we used the four recordings from the two last days of that phase (two recordings per day) as data. The sample size was then 10 samples per group: four recordings from the pre‐experimental phase (no vegetation), and three trials at each vegetation density (low and high vegetation) during the experiment. For each experimental trial, we averaged together the number of contact calls in the five recordings made within 1.5 h. Fixed effects were as follows: (a) the vegetation density (none, low, or high level); (b) the presence/absence of vocalizations from wild white‐eyes; and (c) the proportion of active birds during the 120‐s recording (from 1—all birds active, to 0—no active birds; averaged for all recordings of a trial). We also ran preliminary analyses that included the order of the trial (first, second, and third trial of a treatment) as a fixed explanatory factor; since it was not significant, we removed this factor from the models to avoid issues of over‐specification. We did not include interactions between activity and the other variables because activity was always a strong positive force influencing the number of vocalizations; however, we did include an interaction between vegetation density and the presence of wild white‐eyes. The random factor was the group number. This experiment was conducted on five white‐eye groups.

Due to the small sample size of this analysis (*N* = 47 samples, there were three mistrials), we also conducted a supplementary analysis that used all the 131 recordings made during the experimental phase, but excluded the pre‐experimental recordings without vegetation. The fixed effects were the same as in the above model, with the exception that vegetation now had two levels (high and low); the random effect for these models was the trial in which the five successive recordings were made.

### 2.11 Group effect experiment

As the response variable in these experiments, we used the mean individual‐level contact call rate (total number of calls divided by the numbers of individuals in the subgroup). To incorporate the data when the whole group was present (eight birds; we excluded the groups with only seven birds), we used the 120‐s recordings made twice a day during the two last days of pre‐experimental phase, dividing the total counts of contact calls by eight. The sample size was 13 samples per group: four 120‐s recordings of the whole group (eight birds), with three trials each of three treatments (one, two, four birds; again, results from the five 120‐s recordings made within 1.5 h were averaged together). The fixed effect of interest was here (a) the group size (one, two, four, and eight birds); the other fixed factors were (b) the presence of wild white‐eyes, and (c) proportion of active birds, similar to the vegetation experiment (the order of the trial was again inconsequential and not included). An interaction was included between group size and the presence of wild white‐eyes. The random factor was the group number. This experiment was conducted on 10 white‐eye groups.

In a manner similar to the vegetation effect analysis, we were again concerned by the small sample size (111 samples; 12 recordings in the pre‐experimental phase were removed for three groups that had seven individuals, and there were seven other mistrials), and we conducted a supplemental analysis using all recordings made during the experimental phase (411 recordings made of subgroup sizes of one, two, and four birds). Models had the same fixed factors as above, though group size now had only three levels, and the random factor was identity of the subgroup (i.e., the unique combinations of individuals for each trial).

A final analysis incorporated the social characteristics of the individuals in the subgroups of two and four individuals during the trials of the experimental phase. These models had the same fixed factors as the earlier described group size models, with three additional fixed factors: (d) male ratio (from 1—all male birds; to 0—all female); (e) rank distance (the distance in rank between birds, averaged for the subgroup of four among all pairs of individuals); and (f) affiliation index (also averaged for the subgroup of four). The random factor was the identity of the subgroup.

## 3 RESULTS

### 3.1 Acoustic comparison of different kinds of contact calls

**FIGURE 2:**
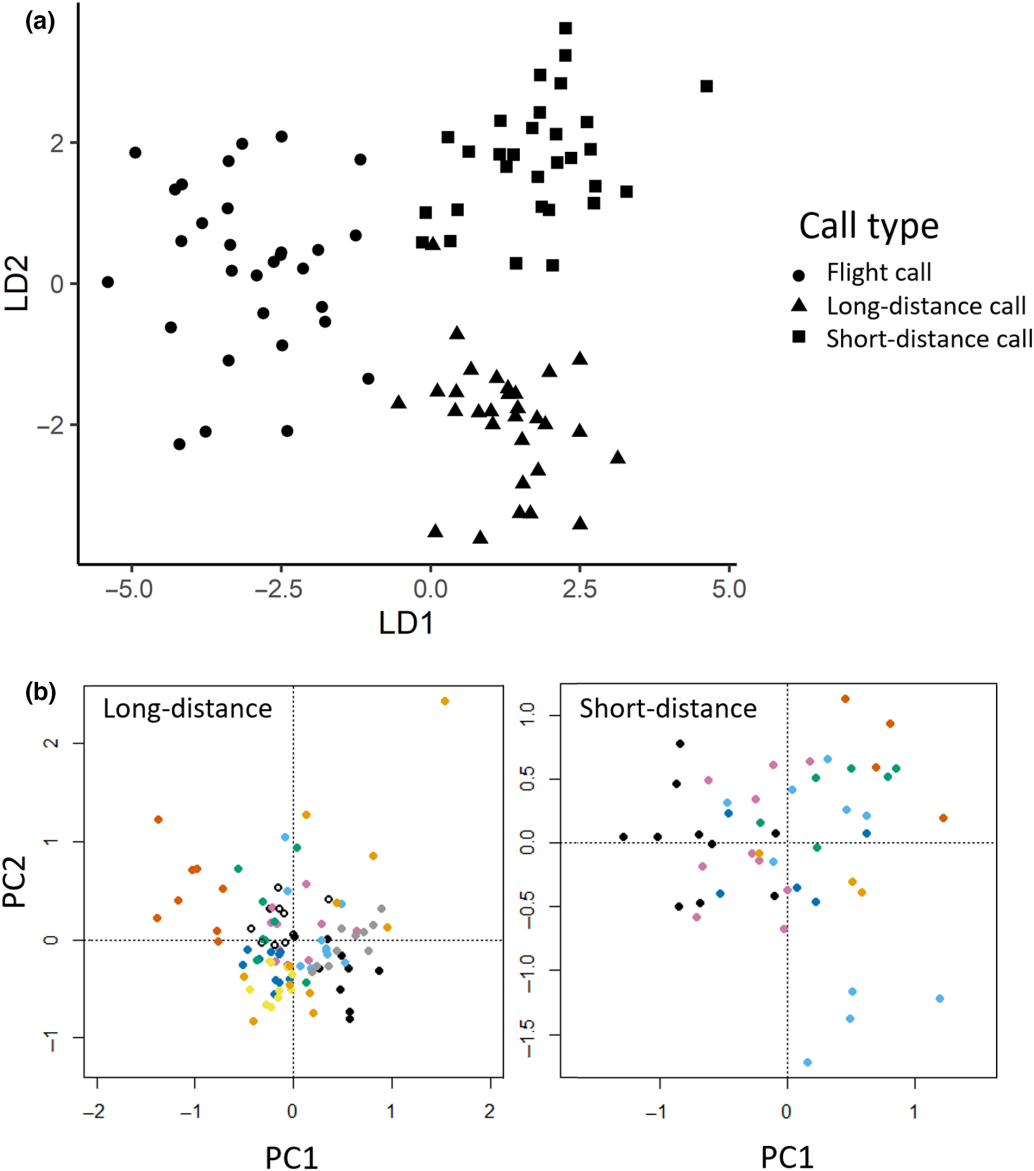
(a) Predictions from Linear Discriminant Analysis (LDA) model plotted on two dimensions. The LDA model was built with the type of contact call as response variable (FC—flight call; LD—long‐distance call; SD—short‐distance call) and the following acoustic parameters as fixed effects: duration (in s), peak frequency, frequency modulation, and midpoint frequency bandwidth (all in Hz). The overall pattern was of the note types being discrete from each other. *N* = 90 notes. (b) The PCA biplots for long‐distance (LD) and short‐distance (SD) calls (respectively, *N* = 96 and 48 notes). Individuals are represented by different colors. The overall pattern for both call types was that notes from the same individual clustered together.

The three kinds of contact calls—short‐distance, long‐distance, and flight call—had different mean rates of production (respectively: 0.70, 0.08, and 0.67 calls per second; *N* = 118 recordings from pre‐experimental phase), and they were distinct in their acoustic characteristics (Figure 1; Table 1). The long‐distance call was characterized by a longer duration compared with the short‐distance and flight calls, and a higher amplitude. Short‐distance and long‐distance contact calls both descended sharply in frequency, whereas the flight call had a more burst‐like shape with less frequency modulation, but a larger midpoint bandwidth than the two other types. Flight calls were also repeated in series, with the first call being longer in duration than the subsequent calls (Figure 1a). The LDA correctly classified 97.8% of observations (*N* = 90 notes; Figure 2a).

**TABLE 1:** Acoustic comparison of the three contact call types: short‐distance, long‐distance, and flight calls.

Call type	Duration (s)	Peak Freq. (Hz)	Peak amp. (U)	Freq. Modulation (Hz)	Freq. Bandwidth (Hz)
Short‐distance	0.15 ± 0.02	4891 ± 461	1222 ± 1393	1662 ± 361	584 ± 324
Long‐distance	0.27 ± 0.03	4831 ± 373	7260 ± 9851	1928 ± 533	509 ± 305
Flight	0.19 ± 0.05	5653 ± 416	2210 ± 3256	984 ± 408	1091 ± 244

*Note*: Here are shown the mean and standard deviation of: duration of the whole note (in s); peak frequency of the first 0.02 s of the note in Hz; peak amplitude of the whole note (in U, a dimensionless unit of amplitude); frequency modulation (peak frequency of the first 0.02 s minus the peak frequency of the last 0.02 s) in Hz; midpoint frequency bandwidth (frequency surpassed by 5% of the note's amplitude minus the frequency surpassed by 95% of the note's amplitude) sampled for 0.02 s in the middle of the note, in Hz. *N* = 30 notes for each call type (three notes sampled for each of the 10 white‐eye groups, taken randomly from the sound files recorded during the pre‐experimental phase).

Long‐distance calls of the wild white‐eyes were of much lower amplitude (570 ± 1539 U, a dimensionless unit of amplitude, *N* = 30 notes) than the long‐distance calls of the captive birds (7260 ± 9851 U, *N* = 30 notes; one‐tailed *t*‐test for independent samples: *t*_58_ = 3.61, *p* < .001). In fact, wild white‐eye calls were usually undetectable in the waveform, although visually apparent in the spectrogram.

### 3.2 Individual differences in acoustic properties of contact calls

The birds housed solitarily were often quiet, especially in making short‐distance calls. This resulted in only 10 individuals included in the analysis for the long‐distance calls, and seven individuals for the short‐distance calls. Two PCA biplots were created separately for long‐distance calls and short‐distance calls (Figure 2b), which showed that the notes produced by the same individuals clustered together. For the long‐distance calls, the first and second axes explained, respectively, 35.2% and 21.1% of the data. The ANOVA on the first axis showed that the individuals diverged strongly in their acoustic characteristics (*F*_9,86_ = 19.47, *p* < .001). For short‐distance calls, the first and second axes explained, respectively, 36.4% and 21.6% of the data. Again, the ANOVA on the first axis showed a clear acoustic divergence between individuals (*F*_6,41_ = 11.14, *p* < .001).

### 3.3 Vegetation effect experiment

We collected 131 recordings during the experimental phase, including 67 for the low‐density vegetation test, and 64 for the high‐density test. In these recordings, there was a total of 26,825 contact call notes. There was a total of 20 recordings without any vegetation during the pre‐experimental phase, which included 3834 contact call notes. When the five recording sessions for one trial during the experimental phase were averaged together, there were 14 low‐density trials and 13 high‐density trials.

The group‐level rates of all of the call types were not influenced by vegetation but were increased by activity level (*Z* > 2.08, *p* < .038; see Table 2). The results of the supplemental analysis that only focused on the experimental phase were similar (Table S2 in Appendix S1): Vegetation density did not affect the group‐level rates of any types of calls, and activity level increased the rates of all call types in this analysis (Z ≥ 3.43, *p* < .001); in addition, the presence of wild white‐eyes increased long‐distance calls (*Z* = 4.37, *p* < .001).

**TABLE 2:** Regression models investigating the effect of vegetation on the group‐level rate of the short‐distance (SDC), long‐distance (LDC), and flight calls (FC).

Call	Mg. *R* ^2^	Variables	Estimate	SE	*Z*	*p*
SDC	0.22	Density (Low)	−0.03	0.40	−0.07	.946
		Density (No)	−0.01	0.32	−0.02	.986
		WildCalls (presence)	0.05	0.34	0.15	.880
		Activity	1.14	0.35	3.27	.001
		Density (Low):WildCalls	−0.04	0.47	−0.09	.929
		Density (None):WildCalls	0.10	0.45	0.23	.822
FC	0.64	Density (Low)	0.30	0.48	0.63	.529
		Density (No)	0.19	0.41	0.46	.646
		WildCalls (presence)	0.36	0.43	0.83	.406
		Activity	3.32	0.59	5.60	<.001
		Density (Low):WildCalls	−0.06	0.56	−0.11	.909
		Density (None):WildCalls	0.13	0.55	0.24	.810
LDC	0.37	Density (Low)	−0.01	0.97	−0.01	.998
		Density (No)	−0.47	0.81	−0.58	.563
		WildCalls (presence)	1.18	0.77	1.53	.126
		Activity	1.69	0.81	2.08	.038
		Density (Low):WildCalls	−0.14	1.07	−0.14	.893
		Density (None):WildCalls	0.36	0.99	0.36	.716

*Note*: There were three predictors (Density—high‐, low‐, and no‐vegetation; Activity—proportion of active birds; WildCalls—presence/absence of wild white‐eyes vocalizing), as well as the interaction between WildCalls and Density. All models were negative binomial GLMMs, with the group number as random factor. The reference categories were high density of vegetation and the absence of wild calls. Mg. *R* ^*2*^ = marginal *R* ^*2*^. *N* = 111 samples.

### 3.4 Group effect experiment

We collected 411 audio recordings during the experimental phase, including 135 made with only one subject, 139 made with two individuals, and 137 with a group of four birds. In these recordings, there was a total of 11,855 contact call notes. There were 28 recordings with eight individuals (the whole group) during the pre‐experimental phase, which included 5009 contact call notes. When the five recording sessions for one trial during the experimental phase were averaged together, there were 27 trials with one individual, 28 trials with two individuals, and 28 trials with four individuals.

**FIGURE 3:**
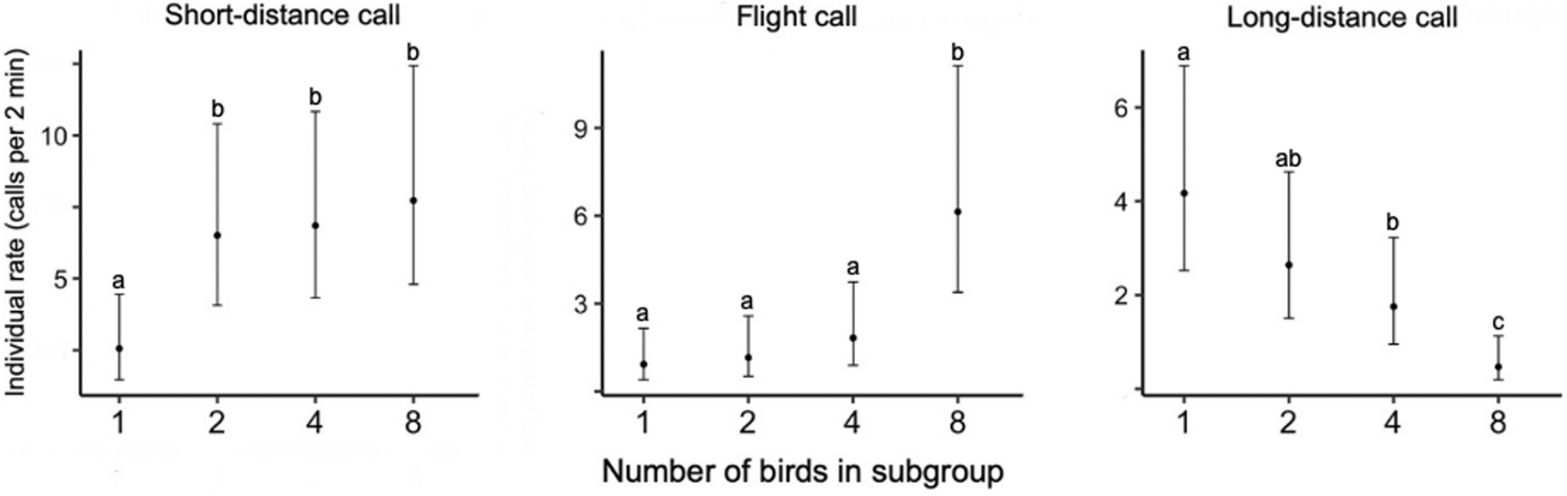
Predicted values of the rate of short‐distance, long‐distance, and flight calls for the different subgroup sizes, including the groups of eight birds recorded in the last 2 days of the pre‐experimental phase. All models are negative binomial GLMMs. *N* = 111 samples.

In the results comparing different group sizes, the mean individual‐level rate of both short‐distance and flight calls increased with increasing group size (Figure 3; Table 3). Short‐distance calls increased with group size, with the one‐bird treatment being lower than all other treatments (*Z* > 2.55, *p* < .011; Tukey's tests: *p* ≤ .002). Flight calls increased with group size, with the eight‐bird treatment higher than all other group sizes (*Z* = 3.95, *p* < .001; Tukey's tests: *p* < .006). In contrast, long‐distance calls decreased with increasing group size, with the one‐bird treatment being smaller than the four‐bird and eight‐bird trials (*Z* < −2.12, *p* ≤ .034; Tukey's tests: *p* ≤ .014). Long‐distance call production also increased with the presence of wild white‐eyes (*Z* = 2.07, *p* = .039). Activity level increased the individual‐level rate of all call types (*Z* > 4.60, *p* < .001). The results of the supplemental analysis that only focused on the experimental phase were similar (see Table S3 and Figure S3 in Appendix S1), although long‐distance calls were not affected by group size in these models.

**TABLE 3:** Regression models investigating the effect of group size and composition on the individual‐level rate of the short‐distance (SDC), long‐distance (LDC), and flight calls (FC).

Call	*R* ^2^ (Cd.–Mg.)	Variables	Estimate	SE	*Z*	*p*	Tukey for group size	
							Test	*p*
SDC	0.63–0.37	Subgroup2	0.97	0.38	2.55	.011		
		Subgroup4	1.31	0.34	3.88	<.001	1–2	.002
		Subgroup8	1.59	0.32	4.97	<.001	1–4	<.001
		WildCalls (presence)	0.16	0.41	0.38	.703	1–8	<.001
		Activity	1.42	0.31	4.60	<.001	2–4	.999
		Subgroup2:WildCalls	−0.08	0.49	−0.16	.876	2–8	.901
		Subgroup4:WildCalls	−0.71	0.48	−1.49	.138	4–8	.941
		Subgroup8:WildCalls	−1.03	0.48	−2.12	.034		
FC	0.74–0.61	Subgroup2	0.06	0.70	0.09	.930		
		Subgroup4	0.72	0.60	1.21	.229	1–2	.891
		Subgroup8	2.20	0.56	3.95	<.001	1–4	.392
		WildCalls (presence)	−0.38	0.71	−0.53	.597	1–8	<.001
		Activity	3.89	0.72	5.42	<.001	2–4	.835
		Subgroup2:WildCalls	0.54	0.94	0.57	.566	2–8	<.001
		Subgroup4:WildCalls	−0.05	0.89	−0.05	.959	4–8	.006
		Subgroup8:WildCalls	−0.21	0.90	−0.24	.811		
LDC	0.68–0.54	Subgroup2	−0.21	0.43	−0.48	.633		
		Subgroup4	−1.03	0.49	−2.12	.034	1–2	.465
		Subgroup8	−2.35	0.62	−3.77	<001	1–4	.014
		WildCalls (presence)	0.79	0.38	2.07	.039	1–8	<.001
		Activity	2.42	0.43	5.68	<.001	2–4	.346
		Subgroup2:WildCalls	−0.33	0.53	−0.62	.536	2–8	<.001
		Subgroup4:WildCalls	0.24	0.59	0.42	.68	4–8	.026
		Subgroup8:WildCalls	0.29	0.82	0.36	.721		

*Note*: There were three predictors (Subgroup—subgroup sizes 1/2/4/8 birds; Activity—proportion of active birds; WildCalls—presence/absence of wild white‐eyes vocalizing), as well as the interaction between WildCalls and Subgroup. The group size 8 was recorded during the pre‐experimental phase. All models were negative binomial GLMMs, with the group number as random factor, and post hoc Tukey's tests conducted between group sizes. The reference categories were group size 1 (Subgroup1) and the absence of wild calls. Both conditional (Cd.) and marginal (Mg.) *R* ^*2*^ are presented. *N* = 111 samples.

**FIGURE 4:**
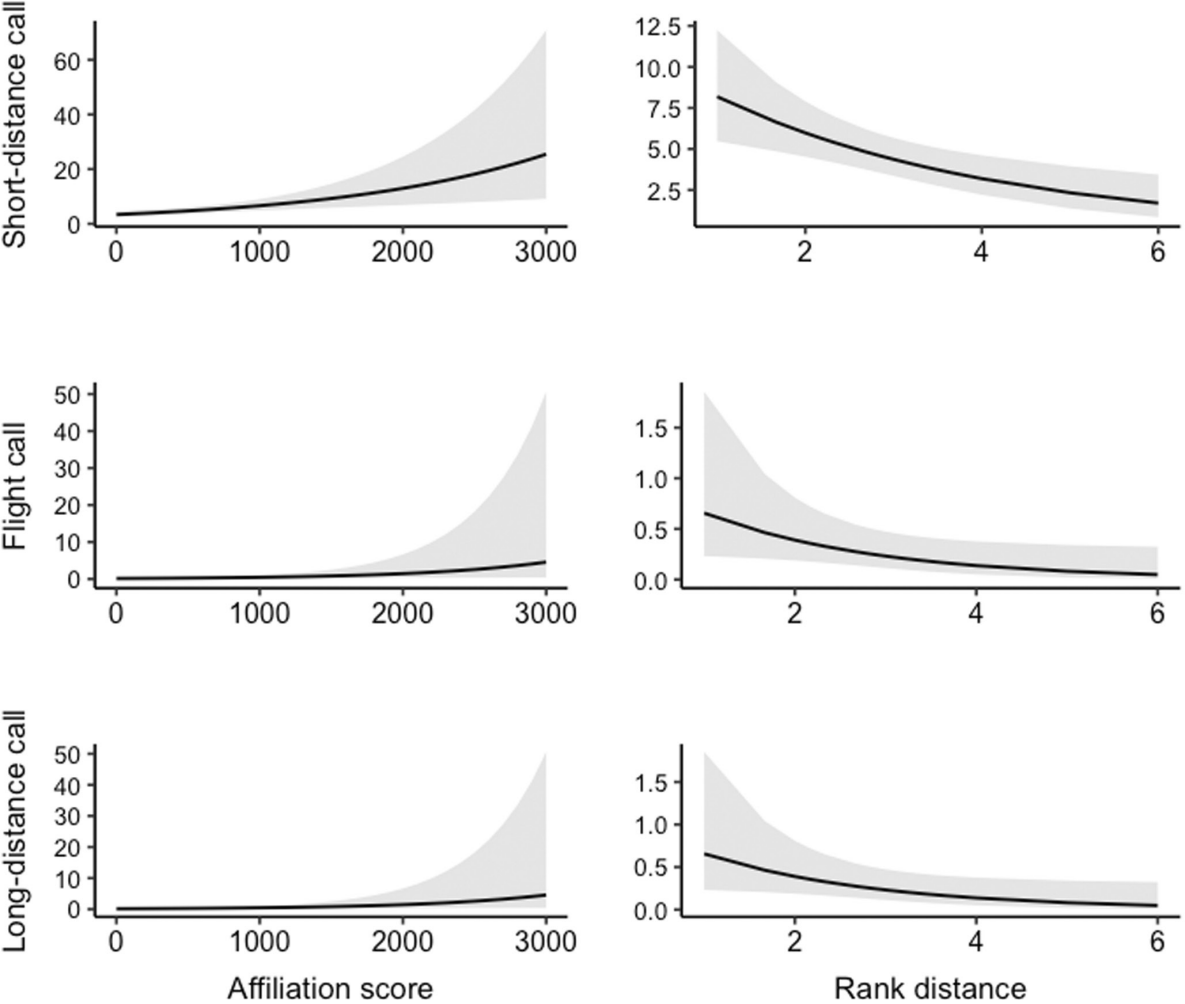
Predicted values of the rate of short‐distance, long‐distance, and flight calls for the social factors of Affiliation score (calculated between pairs of birds as time spent as near neighbors) and Rank distance (based on the social hierarchies inferred using the normalized David's score method). All models are negative binomial GLMMs. *N* = 276 recordings.

When investigating the social factors of dominance, affiliation, and sex‐ratio of the subgroup composition (calculable only for two‐bird and four‐bird treatments), the models showed that all call types decreased with greater distance in dominance rank between birds (*Z* < −2.10, *p* ≤ .036; see Table 4 and Figure 4). Both short‐distance and flight call rate increased with affiliation, though the effect was less strong for flight calls (*Z* = 2.41, *p* = .016), than for short‐distance calls (*Z* = 3.27, *p* = .001). The sex‐ratio only affected the long‐distance call, with a decrease in the individual‐level rate with more males in the subgroup (*Z* = −2.38, *p* = .017). Finally, activity always had a positive effect for all call types (*Z* ≥ 5.84, *p* < .001).

**TABLE 4:** Regression models comparing group sizes of 2 and 4 birds, investigating the effect of social context on the individual‐level rate of the short‐distance (SDC), long‐distance (LDC), and flight calls (FC).

Call	*R* ^2^ (Cd.–Mg.)	Variables	Estimate	SE	*Z*	*p*
SDC	0.58–0.30	Activity	1.18	0.19	6.20	<.001
		Affiliation	<0.01	<0.01	3.27	.001
		Sex ratio	−0.31	0.35	−0.89	.373
		Rank distance	−0.34	0.10	−3.41	<.001
FC	0.78–0.40	Activity	3.41	0.50	6.76	<.001
		Affiliation	<0.01	<0.01	2.41	.016
		Sex ratio	−0.84	0.87	−0.97	.331
		Rank distance	−0.55	0.26	−2.10	.036
LDC	0.50–0.22	Activity	1.68	0.29	5.84	<.001
		Affiliation	−< 0.01	<0.01	−0.19	.852
		Sex ratio	−1.19	0.50	−2.38	.017
		Rank distance	−0.33	0.14	−2.35	.019

*Note*: Three predictors were shared with other models (Subgroup—subgroup sizes 2 and 4 birds; Activity—proportion of active birds; WildCalls—presence/absence of wild white‐eyes vocalizing), as well as the interaction between WildCalls and Subgroup (results for these factors were similar to Table S3). Additionally, other fixed effects for these models about social factors were Affiliation (affiliation scores between pairs of individuals), Sex ratio and Rank distance; all averaged for subgroup of four birds. Only these social variables are shown here. All models were negative binomial GLMMs, with the identity of the subgroup as random factor. Both conditional (Cd.) and marginal (Mg.) *R* ^*2*^ are presented. *N* = 276 recordings.

## 4 DISCUSSION

We did not find evidence in white‐eyes that group‐level contact calling is maintained at a specific rate: White‐eyes did not increase their individual call rate when in smaller groups, at least for the most common contact call types (short‐distance and flight call; see below for a discussion of the rarer long‐distance call). To the contrary, individual‐level call rates for these call types increased with group size, and the group‐level rates for large groups were thus much higher than for small groups. This result does not support the hypothesis that the group‐level rate of contact calls could function as an indication of immediate predation risk through its cessation, because the group‐level rates were low for small groups and thus silence would not be as perceptible. Nor did we find that call rate was influenced by another kind of environmental stimulus, the density of vegetation. Rather, social stimuli, and specifically the presence of affiliated individuals and individuals of similar levels on the dominance hierarchy, were associated with higher individual‐level calling rates. Together with other studies on Swinhoe's White‐eyes (Meaux, He, et al., 2021; Meaux, Peabotuwage, et al., 2021), these findings suggest that changes in contact call rate in this species are more influenced by social context than by environmental conditions. Below, we discuss in detail the effects of vegetation density, and then the effects of group size and group composition on the different call types.

Our first finding in this study was about vegetation. We found that vegetation density does not have a strong effect on the group‐level contact call rate of the captive birds, in comparing between high density, in which the artificial bamboo formed walls that could not be seen through, and low‐density conditions, or even when there was no vegetation. In the case of the dense vegetation, the group of eight individuals was often split, with some birds on one side of the bamboo walls and others on the other side. Therefore, it appears that visual obstruction between birds did not increase the rate of contact calls (measured at the group level in this experiment, as we did not manipulate the number of individuals). The result is consistent with our work in the field. There, aspects of vegetation density explained only 2% of the variation in the group‐level contact call, which was rather related to movement, specifically increasing before peripheral birds flew into the center of the group (Meaux, Peabotuwage, et al., 2021). The conclusion of the field study was that call rate did not convey information on environmental conditions when both signaller and receiver were relatively close together, although it might function as a recruitment call (see e.g., Gall & Manser, 2017; Mahurin & Freeberg, 2009). In the artificial situation of the aviary, where birds were not continually moving into areas where they had not yet foraged, it was difficult to determine the connection of the birds' calling patterns to recruitment.

Other studies have found mixed results as to whether habitat density affects the production of repetitive vocalizations. Our result is most similar to a study in two species of gorillas, in which vegetation structure was not found to affect contact calls rates (Hedwig et al., 2015). In contrast, olive baboons, *Papio hamadryasi*, showed greater call rates in thicker habitats in one of two study sites (Ey et al., 2009). Sentinel dwarf mongoose called more in denser habitat (Kern & Radford, 2013), and sentinel pied babblers had longer sentinel bouts when visibility was poor (Hollén et al., 2011). Krama et al. (2008) also showed that crested tits, *Parus cristatus*, made more loud contact calls in denser vegetation, but this result may have been influenced by these conspicuous calls attracting predator attention in more open and risky environments (Krams, 2001). Significantly, those studies that also looked at social factors, such as for instance the distance with the nearest neighbor, found evidence that social factors were important as well (Ey et al., 2009; Hedwig et al., 2015; Kern & Radford, 2013).

Our second finding was about group size, and its potential connection to another environmental factor, predation risk. One of our hypotheses for the overall project was that the cessation of contact calls could be a signal of immediate danger. While we recognized that sudden silence might not be easy to attain without all group members detecting a threat, we thought that silence could reinforce the reliability of an alarm signal and that contact calls resuming after an alarm call would indicate that it was a false alarm (Goodale & Kotagama, 2005; Munn, 1986; Wolf et al., 2013). However, an experimental study performed simultaneously to this one demonstrated that group‐level contact call rate did not appear to be related to predation risk (Meaux, He, et al., 2021). Indeed, after exposure to a predator model, the birds did not abruptly cease calling. Furthermore, when we played back contact calls and then ceased that playback, or played back alarm calls followed by silence, the silence did not modulate the birds' response (the birds did respond to alarm calls, but did so strongly even when alarm calls were followed by contact calls).

In the group size experiment presented here, the predation risk hypothesis led us to expect that smaller group sizes would have higher individual‐level rates of calling, so that a cessation of calling would be perceptible. However, only the rate of long‐distance calls showed the pattern in which the overall group‐level rate stayed stable as group size decreased (see Figure 3). Yet, long‐distance calls were relatively rare (2.71 ± [SD] 4.82 calls per minute for a group of eight birds, compared to 42.41 ± [SD] 21.51 for short‐distance calls, and 44.32 ± [SD] 47.95 for flight calls; *N* = 28 notes for each call type) and the cessation of such an infrequent call type could hence not be an obvious signal to indicate danger. Thus, this study supports the idea that contact calling does not function as an indicator of immediate predation risk in this species. A similar finding—a lack of relationship between calling rate and alarm threats—was also shown for another social and vocal species, the zebra finch (Butler et al., 2017).

This study shows that rather than being influenced by habitat density or immediate predation risk, contact call rate is instead shaped by social factors (although we acknowledge that social factors may be in turn related to predation risk at longer time spans—for example, birds may choose to stay close to other group members due to their perceived predation risk). The different kinds of contact calls were differentially influenced by group size. For long‐distance contact calls, individual‐level rate was high for birds in solitary and small group sizes, and quite low for the full group. This seems to indicate that birds might use such calls to communicate with their distant group members that were removed from the aviary (see similar observations in the contact calling of very different organisms: Dahlin et al., 2005; Janik & Slater, 1998; Oda, 1996; Rendall et al., 2000). This is consistent with the fact that loud and long (in duration) calls can be transmitted on a wider ranger than softer calls (Wiley & Richards, 1982). There was another aspect of long‐distance call use. When a wild white‐eye flock was present near the aviary, the individual‐level rate of long‐distance calls increased. Therefore, our data on long‐distance call use are consistent with other bird species that use a louder call type for long‐distance communication within or between groups (Crane et al., 2016; Krama et al., 2008; Sieving et al., 2010).

Short‐distance and flight call rates showed the opposite pattern from long‐distance calls, increasing at the individual level as group size increased. Flight calls, which were only produced in flight, increased rapidly when the full group was in the aviary. We believe this result is directly due to flights increasing with group size, possibly due to more competition at the feeders of the aviary. For short‐distance calls, the largest difference was between the solitary treatment and all other treatments. The lack of short‐distance calls when the birds were solitary suggests that they usually stop producing this call in the absence of an audience.

For both short‐distance and flight calls, individual‐level rate was influenced by the affiliation between individuals, with more calls between birds that often spent time together. Of all the call types, short‐distance calls had the most significant relationships between rate and the social factors (both affiliation and dominance hierarchy). This is consistent with the idea that this call type is used for within‐group communication and has some connection to the relationships between pairs of birds (Harcourt & Stewart, 1996; Levréro et al., 2019). Several bird studies have shown some similar results in the influence of social relationships on call rate. For example, Freeberg and Harvey (2008) found that chickadee pairs that perched closer to each other made more chick‐a‐dee notes. This result was confirmed in an audience effect experiment in which chickadees in groups of familiar individuals called more than when in groups of unfamiliar individuals (Coppinger et al., 2018). Similar results have been shown in budgerigars, *Melapsittacus undulatus* (Striedter et al., 2003) and pinyon jays, *Gymnorhinus cyanocephalus* (Dahlin et al., 2005). In zebra finches, *Taeniopygia guttata*, the group and individual rate of calling actually decreased with the percentage of paired birds in the group (Elie et al., 2011; Fernandez et al., 2017). Primates have also been shown to have contact call types that have specific audiences (Bolt & Tennenhouse, 2017; Rendall et al., 2000; Rukstalis et al., 2003).

For all three call types, individual‐level call rates were lower when birds were housed with individuals of contrasting dominance rank. One general explanation for this result could be that the production of contact calls is influenced by the proximity of group members (Engesser & Manser, 2022): Individuals that are more distant on the social hierarchy might be less spatially close to each other in the aviary, and thus calling might decrease. Alternatively, or additionally, it could also be possible that subordinates in particular lower their call rates in the presence of dominants. Bird flocks, including white‐eyes (Kikkawa, 1980), often show strict dominance hierarchies, with dominant birds showing better body condition and higher survival (Krams et al., 2020; Lahti, 1998), so subordinates might withhold information about their location in order to avoid aggression and interference competition (Wiley, 1983). Analogously, some avian species have been reported to recruit others to food only when competition can be reduced by the resource being divisible (Elgar, 1986), or when the signaller can demonstrate its dominance (Clay et al., 2012). Birds are also known to change their strategies depending on the dominance of other birds they are grouped with (e.g., Bugnyar & Heinrich, 2006). However, in the case of this study, the hypothesis that call rates decreased when birds of contrasting rank were housed together because subordinates called less cannot be directly tested, since we measured call rates at the mean individual level of the whole group, and therefore were unable to see whether indeed lower ranked birds made fewer calls.

Our result that the birds appear to have distinctive individual acoustic characteristics to their contact calls is important in determining what kind of information they may gain by listening to each other. First, we need to acknowledge that we do not show that the birds can use distinctiveness between individuals to recognize each other. Second, our data on this subject are limited, as each individual was only recorded during a 1‐h period. Yet, Robertson (1996) showed in a congeneric species that mates could recognize each other, so we think it is likely that Swinhoe's White‐eye contact calls are also individually recognizable. If we assume for argument's sake that birds can recognize individuals, it would go a long way towards understanding what information birds could gain by listening to contact calls. Even with their visual focus restrained (Whittingham et al., 2004), they could still hear information about where dominants, or their most affiliated partners, are located. They could then withhold calls when a dominant might be eavesdropping, or use an increase in rate to recruit an affiliated individual closer to them. While our data cannot say that contact calls have a recruitment function, the pattern that affiliated birds have higher rates of contact calling when they are together is consistent with this idea.

We need to acknowledge some limitations to this study. First, we had to calculate a mean individual‐level rate (measuring first the group‐level rate, and then dividing by the number of individuals present), as we were unable to track and identify every vocalizing individual, due to the high number of calls produced (1.45 ± [SD] 0.94 per s in a group of 8; *N* = 117 recordings of the whole group), the softness and briefness of the calls, and the number of birds. It would be useful in future research to use a methodology that could track callers, for example, with each bird carrying a small microphone setup (Anisimov et al., 2014; Gill et al., 2016). Concerning the group size experiment, an important point to mention is that stress could be a confounding variable linked to group size. Birds in subgroups of one, two, or four birds had undergone a proceeding phase of capture (experimenters catching other group members with butterfly nets), whereas this had not happened for the groups of eight birds. This could be an aspect in which the behavior we documented was unrepresentative of natural conditions (Webster & Rutz, 2020). However, this effect was reduced by waiting a minimum of 30 minutes after capture to start the recordings (subjects often displayed normal foraging behavior within the first 15 min after excluding their group mates). Stress is unlikely to explain differences between subgroups of two and four birds, and it does not explain the connection of affiliation and dominance rank to contact call rate in these group sizes.

## 5 CONCLUSIONS

Our study showed that contact call rates are most affected by social stimuli, including both group size and the presence of particular individuals. Contact call rate is not affected by vegetation density, nor do birds increase their calling in smaller groups to maintain a high call rate that would make a shift to silence more salient in a predation context. We think this result is consistent with other studies that have shown the contact call rates in birds can fluctuate a lot temporally in different social contexts that vary in movement rates, activity levels or time of day (Elie et al., 2011; Hillemann et al., 2019; Meaux, Peabotuwage, et al., 2021). For example, the “chirrup” call of house sparrows studied by Elgar (1986) varies very strongly in rate, and gets especially high near sundown, as birds form roosting groups (EG personal observation). Our results suggest that contact call rate may change depending on whether birds are willing to disclose, or want to emphasize, their location to certain other group participants. We hope more work can be performed on understanding the relationship between individual‐ and group‐level contact calling, the relationship between the rate of the contact calls and collective decision‐making (Couzin, 2009), and particularly how groups come to a consensus about their movement (Boinski & Garber, 2000; Bousquet et al., 2011; Conradt & Roper, 2005).

## AUTHOR CONTRIBUTIONS

**Estelle Meaux:** Conceptualization (equal); data curation (equal); formal analysis (equal); investigation (equal); methodology (equal); software (equal); visualization (equal); writing – original draft (equal); writing – review and editing (equal). **Chao He:** Investigation (equal); resources (equal). **Xiaolei Zeng:** Investigation (equal); resources (equal). **Ruchuan He:** Investigation (equal); resources (equal). **Aiwu Jiang:** Methodology (equal); project administration (equal); resources (equal). **Eben Goodale:** Conceptualization (equal); funding acquisition (equal); methodology (equal); project administration (equal); supervision (equal); writing – original draft (equal); writing – review and editing (equal).

## FUNDING INFORMATION

This work was supported by the National Natural Science Foundation of China (grant number 31770424) to EG, and a Chinese Government Scholarship to EM.

## CONFLICT OF INTEREST STATEMENT

The authors declare no competing interest.

### OPEN RESEARCH BADGES

This article has earned an Open Data badge for making publicly available the digitally‐shareable data necessary to reproduce the reported results. The data is available at 10.5061/dryad.80gb5mkvd.

## Supporting information


Appendix S1

## Data Availability

The data that support the findings of this study are openly available in the Dryad Digital repository: 10.5061/dryad.80gb5mkvd
