# Supplementary material for: Audience effects in a group‐living bird: How contact call rate is affected by vegetation and group size and composition
Source: Ecol Evol. 2023 Mar 24;13(3):e9909. doi: 10.1002/ece3.9909 (PMC10037432; doi:10.1002/ece3.9909)

**SUPPORTING INFORMATION**

**Audience effects in a group-living bird: How contact call rate is affected by vegetation and group size and composition**

Estelle Meaux^a^* (ORCID number: 0000-0002-5609-2384), Chao He^a^ (0000-0002-8102-9272), Xiaolei Zeng^a^ (0000-0002-1005-8877), Ruchuan He^a^ (0000-0003-4318-9221), Aiwu Jiang^a^ (0000-0002-5259-298X) and Eben Goodale^a^* (0000-0003-3403-2847)

^a^ Guangxi Key Laboratory of Forest Ecology and Conservation, College of Forestry, Guangxi University, Nanning, Guangxi, China

*Correspondence:

Estelle Meaux, Guangxi Key Laboratory of Forest Ecology and Conservation, College of Forestry, Guangxi University, Nanning, Guangxi, China. E-mail: estelle.meaux@outlook.com. Telephone: +447741583784.

Eben Goodale, Guangxi Key Laboratory of Forest Ecology and Conservation, College of Forestry, Guangxi University, Nanning, Guangxi, China. E-mail: ebengoodale@gxu.edu.cn, eben.goodale@outlook.com. Telephone: +8618174128262.

Table of Contents

[Capture method 4](#_Toc121905679)

[Aviary conditions 4](#_Toc121905680)

[Tests of detection of contact calls under aviary conditions 5](#_Toc121905681)

[Acoustic measurements 6](#_Toc121905682)

[Supplementary analyses of vegetation and audience effect 6](#_Toc121905683)

[**Table S1**. Ethogram of affiliative and agonistic behaviours of Swinhoe’s White-eyes in captivity. 8](#_Toc121905684)

[**Table S2**. Regression models investigating the effect of vegetation on the group-level rate of the short-distance (SDC), long-distance (LDC), and flight calls (FC), when only using data from the experimental stage 9](#_Toc121905685)

[**Table S3**. Regression models investigating the effect of group size and composition on the individual-level rate of the short-distance (SDC), long-distance (LDC), and flight calls (FC), when only using data from the experimental stage 10](#_Toc121905687)

[**Figure S1**. Picture of a trapping cage. 11](#_Toc121905689)

[**Figure S2.** A) Photography of the aviary; B) Top view of the map of the aviary. 12](#_Toc121905690)

[**Figure S3**. Predicted values of the rate of short-distance and flight calls for the subgroup sizes 1, 2 and 4 birds 13](#_Toc121905691)

Capture method

Before capture we scouted different areas of Guangxi University campus and surrounding green spaces looking for white-eye groups, until finding areas which they frequented. During these observations we also looked for any evidence of breeding, and avoided capture if we saw any. The main structure of each trapping cage (34 x 22 x 37 cm) is made of wood, and the bars are made of thin wooden stems (Fig. S1). A large hook at the top of the cage allows it to be placed on small tree branches, using a telescopic pole of 4 m length. In one compartment of the cage a living Swinhoe’s White-eye belonging to the previously tested group was placed as a decoy. Five traps were then placed in the subcanopy to canopy of small trees in an area of approximately 10 m radius. Birds that entered the trap were unable to get out, and they were removed from the trap when we checked on them every hour. Food and water were provided for both decoy and trapped birds. A capture event would take place within a day. At capture, birds were checked for brood patches, and if they showed such evidence of breeding, or were juvenile, or did not appear to be in good body condition, they were immediately released. If we trapped more than eight birds, we released some extra individuals; on the other hand, if we trapped less than seven birds, all captured individuals were released before searching another location to catch a different flock.

Aviary conditions

The outdoor aviary (Fig. S2A) is located in the north area of the Guangxi University campus. It is made with galvanized wire mesh (mesh size 1 cm^2^) with the roof being a translucent polycarbonate panel, which protects from direct sunlight without completely obscuring light. In addition to the main cage, there is an entrance room, which serves to make sure birds do not escape when experimenters enter and exit, and an adjacent working room that has a 1.5-m square window allowing direct observation of the aviary, as well as audio recording when it was opened (Fig. S2B). Several perches (tree branches of length of 0.5-1 m with leaves attached) were placed randomly inside the aviary. Before each new flock was introduced perches were replaced and the concrete floor was cleaned. During the captivity period, the birds were provided with water *ad libitum* in water dispensers as well as in a large, shallow plastic tray that allowed them to bathe. Moreover, sprinklers were activated at regular times throughout the day to create droplets on leaves. Diverse fruits, living mealworms and a mixture of seeds and cereals were provided *ad libitum* for this omnivorous species, and spread over several feeders, hung between 1-2 m in height to avoid spatial competition. During some times, when experiments on foraging behaviour were being conducted, birds were mostly fed in boxes on the ground.

Prior to release, birds were weighed, measured (culmen, wing, tarsus and tail length), and blood sampled (approximately 0.1cc of blood was sampled from the brachial vein with a sterile 30-gauge needle and a capillary tube). Blood samples were stored in microvials at -80°C, and then sent to a laboratory for chromosomal sex determination.

Tests of detection of contact calls under aviary conditions

To test whether we could detect all contact calls produced from the work room, we first measured their call volume with a sound level meter (CEM model DT-805), using its fast, A frequency weighting options. When the sound level meter was positioned 3.5 m from the birds (with the birds active and in random positions vis-à-vis their orientation towards the observer), the average amplitude of calls was 49.5 ± [SD] 2.5 dB (*N* = 20), ranging from 45.0 to 53.6 dB. Then we created a sound file with a variety of high-quality exemplars of different calls (short- and long-distance calls, flight calls, alarm calls). This sound file was played through a speaker placed at the farthest end of the aviary away from the work room, with the volume of the speaker previously calibrated using the sound meter to reproduce calls between 45 and 47 dB at 3.5 m. Finally, recordings of these trials were scored to see if we could detect all the calls on the sound file, using Raven, constructing spectrograms with 512 FFT and a Hann Window. With a spectrum view, we calculated for each call on the sound file the difference in dB between the background noise and the peak amplitude. The calls had amplitude of 24.4 ± [SD] 7.0 dB (*N* = 30), demonstrating that all the different note types that the birds could make could be detected with our microphone set-up.

Acoustic measurements

We designed our acoustic measurements so as to best distinguish between frequency modulation (frequency change over time) of the whole note and tonality (as measured by frequency bandwidth) at any one point in the note. Our first set of measurements was developed in order to distinguish between call types, and therefore included amplitude. We measured: (a) duration of the whole note (in s); (b) peak frequency of the beginning 0.02 s of the note (in Hz; we selected the beginning of the note because white-eye calls start with high amplitude, and so we could measure the frequency at that point most consistently); (c) frequency modulation (peak frequency of the beginning 0.02 s minus the peak frequency of the end 0.02 s, in Hz); (d) the midpoint frequency bandwidth, defined as the difference between the frequency surpassed by 5% of the note’s amplitude and the frequency surpassed by 95% of the note’s amplitude, sampled for 0.02 s in the middle of the note (in Hz; we selected the midpoint of the note because bandwidth was largest there); and (e) the peak amplitude of the whole note, measured from the waveform (in kU, a dimensionless unit of amplitude).

Our second set of measurements was developed to look at differences between individuals. Because we were not comparing note types, we did not include amplitude in these six measurements. The (a) duration of the note and the (b) frequency modulation were measured in the same way as above. The other frequency measurements were made in a slightly more general way because they were used on two concurrent projects, one of which was on vocalizations that were not from white-eyes. They were: (c) peak frequency assessed over the whole note, (d) maximum frequency in Hz of the whole note, measured using Raven’s “95% frequency” measurement tool, (e) minimum frequency of the whole note in Hz (using the “5% frequency” tool); and (f) frequency bandwidth for the whole note.

Supplementary analyses of vegetation and audience effect

As mentioned in the main text, Tables S1 and S2 and Figure S3 show results of analyses that use only trials from the experimental period, in contrast to the models shown in the main text, which include data from the pre-experimental period. The advantage in using only experimental period trials is that each recording can be a datum, and hence sample sizes are larger (in contrast, in the main text, the information from five trials is averaged together, so that it can be better compared to the pre-experimental stage information).

Table S1. Ethogram of affiliative and agonistic behaviours of Swinhoe’s White-eyes in captivity.

| Behaviour | Definition |
| --- | --- |
| *Agonistic* |  |
| Threatening | opens widely the beak with a movement of the head towards an individual, often with wings shaking |
| Displacing | moves rapidly towards an individual causing the receiver to be relocated, often while threatening (beak opened) |
| Chasing | first displaces an individual then flies after him/her for a short period of time while the receiver tries to flee |
| Pecking | slight but quick movement of the beak (closed) towards another individual, often touching this individual |
| Biting | grasps the feather or the flesh of a neighbour with the beak |
| Pushing | jumps on another individual to push him/her using the legs |
|  |  |
| *Affiliative* |  |
| Physical contact | two individuals touching each other, often while resting or allopreening |
| Allopreening | licks the body (feather and occasionally legs) of another individual |
| Co-feeding | two individuals eating in the same feeder or the same fruit |
| Food transferring | gives food to another individual, sometimes directly transferring the food from beak to beak |

Table S2. Regression models investigating the effect of vegetation on the group-level rate of the short-distance (SDC), long-distance (LDC), and flight calls (FC)

, when only using data from the experimental stage. There were three predictors (Density = high- and low-density of vegetation; Activity = proportion of active birds during the test; WildCalls = presence/absence of wild white-eyes vocalizing during test), as well as the interaction between WildCalls and Density. The random factor was the trial number. All models are negative binomial GLMMs. Both conditional (Cd.) and marginal (Mg.) *R^2^* are presented. *N* = 131 recordings.

| Call | R^2^ (Cd. – Mg.) | Variables | Estimate | S.E. | Z | P |
| --- | --- | --- | --- | --- | --- | --- |
| SDC | 0.80 – 0.23 | Density(low) | -0.13 | 0.24 | -0.57 | 0.57 |
|  |  | WildCalls(presence) | 0.26 | 0.17 | 1.52 | 0.13 |
|  |  | Activity | 1.57 | 0.29 | 5.38 | <0.001 |
|  |  | Density:WildCalls | -0.29 | 0.22 | -1.36 | 0.175 |
| FC | 0.75 – 0.15 | Density(low) | 0.06 | 0.45 | 0.13 | 0.894 |
|  |  | WildCalls(presence) | 0.24 | 0.24 | 0.97 | 0.332 |
|  |  | Activity | 1.78 | 0.52 | 3.43 | <0.001 |
|  |  | Density:WildCalls | -0.22 | 0.26 | -0.84 | 0.401 |
| LDC | 0.43 – 0.36 | Density(low) | -0.78 | 0.44 | -1.77 | 0.077 |
|  |  | WildCalls(presence) | 1.74 | 0.40 | 4.37 | <0.001 |
|  |  | Activity | 2.21 | 0.64 | 3.43 | <0.001 |
|  |  | Density:WildCalls | 0.53 | 0.56 | 0.95 | 0.345 |

Table S3. Regression models investigating the effect of group size and composition on the individual-level rate of the short-distance (SDC), long-distance (LDC), and flight calls (FC)

, when only using data from the experimental stage. There were three predictors (Subgroup = subgroup sizes of 1, 2 and 4 birds; Activity = proportion of active birds; WildCalls = presence/absence of wild white-eyes vocalizing), as well as the interaction between WildCalls and Density. All models are negative binomial GLMMs, with subgroup identity as random factor. The reference category is group size 1 (Subgroup1). Post-hoc Tukey tests between group sizes were conducted (n.a. = not available, when the variable Subgroup was not retained after simplification). Both conditional (Cd.) and marginal (Mg.) *R^2^* are presented. *N* = 411 recordings.

| Call | R^2^ (Cd. – Mg.) | Variables | Estimate | S.E. | Z | P | Tukey  for group size | |
| --- | --- | --- | --- | --- | --- | --- | --- | --- |
|  |  |  |  |  |  |  | Test | P |
| SDC | 0.54 – 0.27 | Subgroup2 | 1.48 | 0.31 | 4.71 | <0.001 | 1 – 2 | <0.001 |
|  |  | Subgroup4 | 1.73 | 0.31 | 5.51 | <0.001 |  |  |
|  |  | WildCalls(presence) | 0.25 | 0.41 | 0.60 | 0.546 | 1 – 4 | <0.001 |
|  |  | Activity | 1.14 | 0.18 | 6.24 | <0.001 |  |  |
|  |  | Subgroup2:WildCalls | -0.41 | 0.46 | -0.88 | 0.377 | 2 – 4 | 0.693 |
|  |  | Subgroup4:WildCalls | -0.47 | 0.47 | -1.00 | 0.316 |  |  |
| FC | 0.57 – 0.27 | Subgroup2 | 1.68 | 0.66 | 2.54 | 0.011 | 1 – 2 | 0.047 |
|  |  | Subgroup4 | 2.46 | 0.65 | 3.81 | <0.001 |  |  |
|  |  | WildCalls(presence) | 0.47 | 0.82 | 0.57 | 0.567 | 1 – 4 | 0.001 |
|  |  | Activity | 2.47 | 0.40 | 6.13 | <0.001 |  |  |
|  |  | Subgroup2:WildCalls | -0.52 | 0.91 | -0.57 | 0.568 | 2 – 4 | 0.328 |
|  |  | Subgroup4:WildCalls | -0.67 | 0.93 | -0.72 | 0.472 |  |  |
| LDC | 0.45 – 0.18 | Subgroup2 | 0.13 | 0.37 | 0.36 | 0.720 | 1 – 2 | 0.821 |
|  |  | Subgroup4 | -0.58 | 0.38 | -1.50 | 0.134 |  |  |
|  |  | WildCalls(presence) | 0.05 | 0.35 | 0.14 | 0.886 | 1 – 4 | 0.822 |
|  |  | Activity | 1.84 | 0.25 | 7.24 | <0.001 |  |  |
|  |  | Subgroup2:WildCalls | 0.16 | 0.47 | 0.35 | 0.727 | 2 – 4 | 0.428 |
|  |  | Subgroup4:WildCalls | 0.71 | 0.50 | 1.43 | 0.153 |  |  |

Figure S1. Picture of a trapping cage.


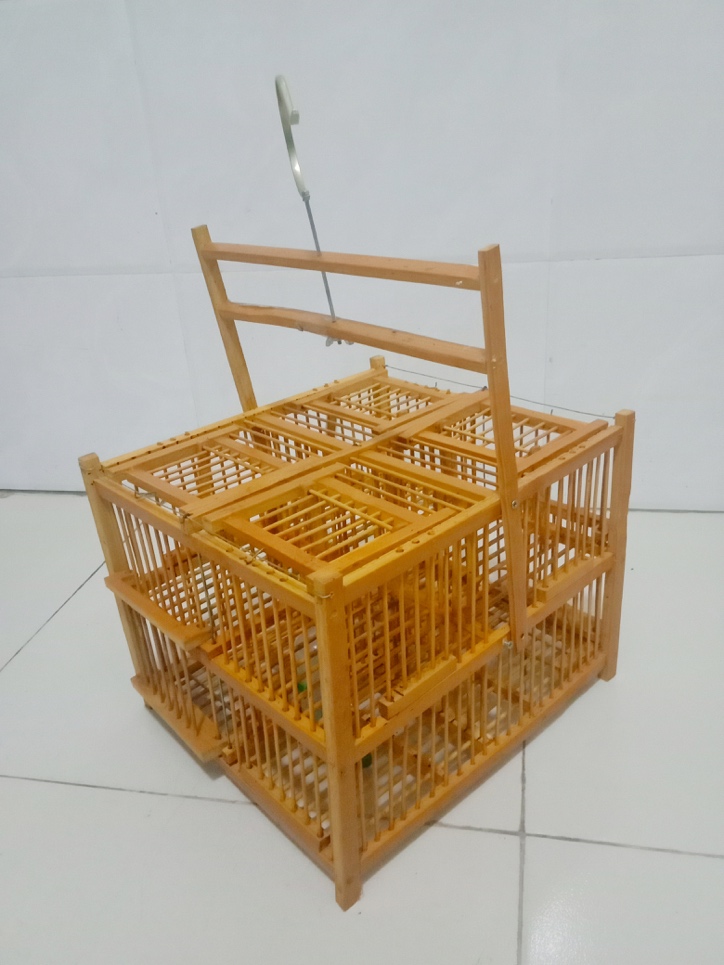


Figure S2. A) Photography of the aviary; B) Top view of the map of the aviary.

**A
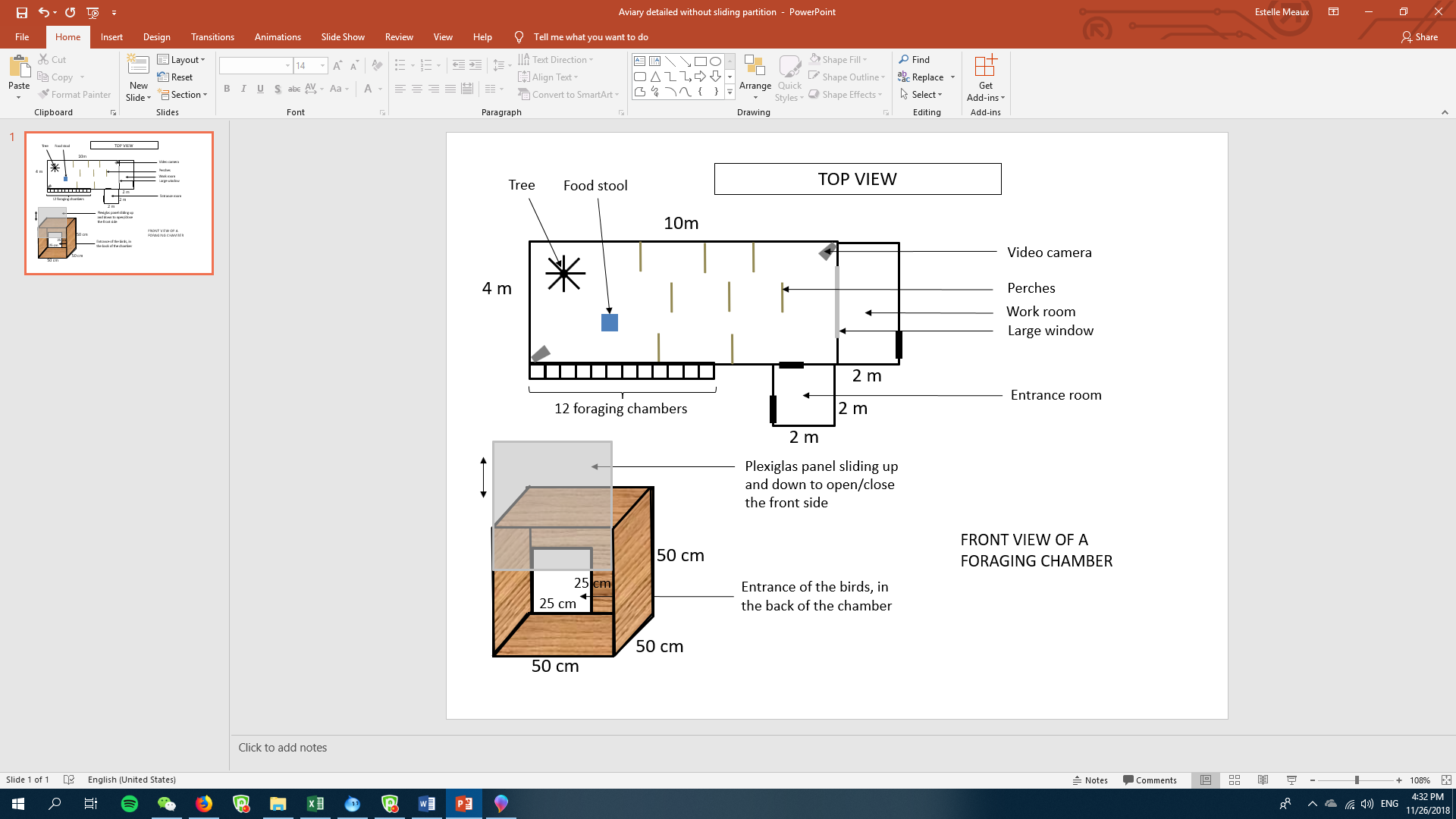
**


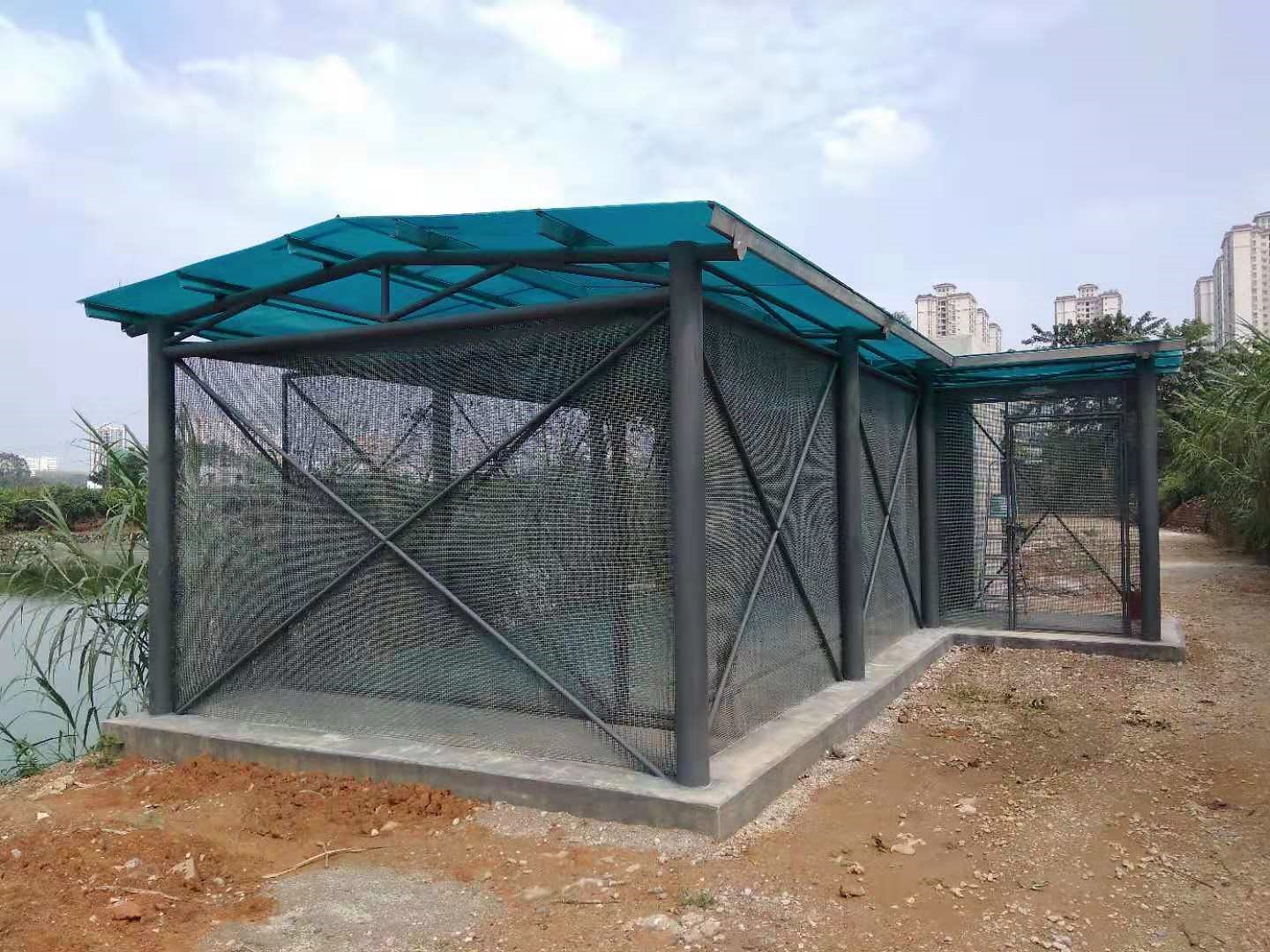


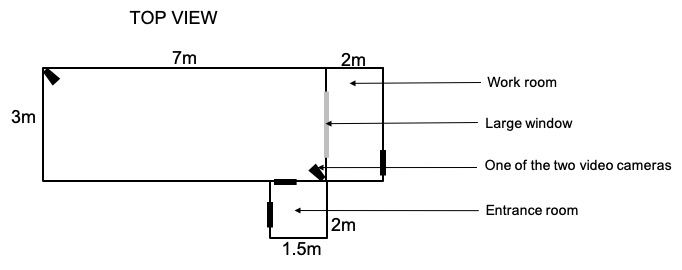


**B
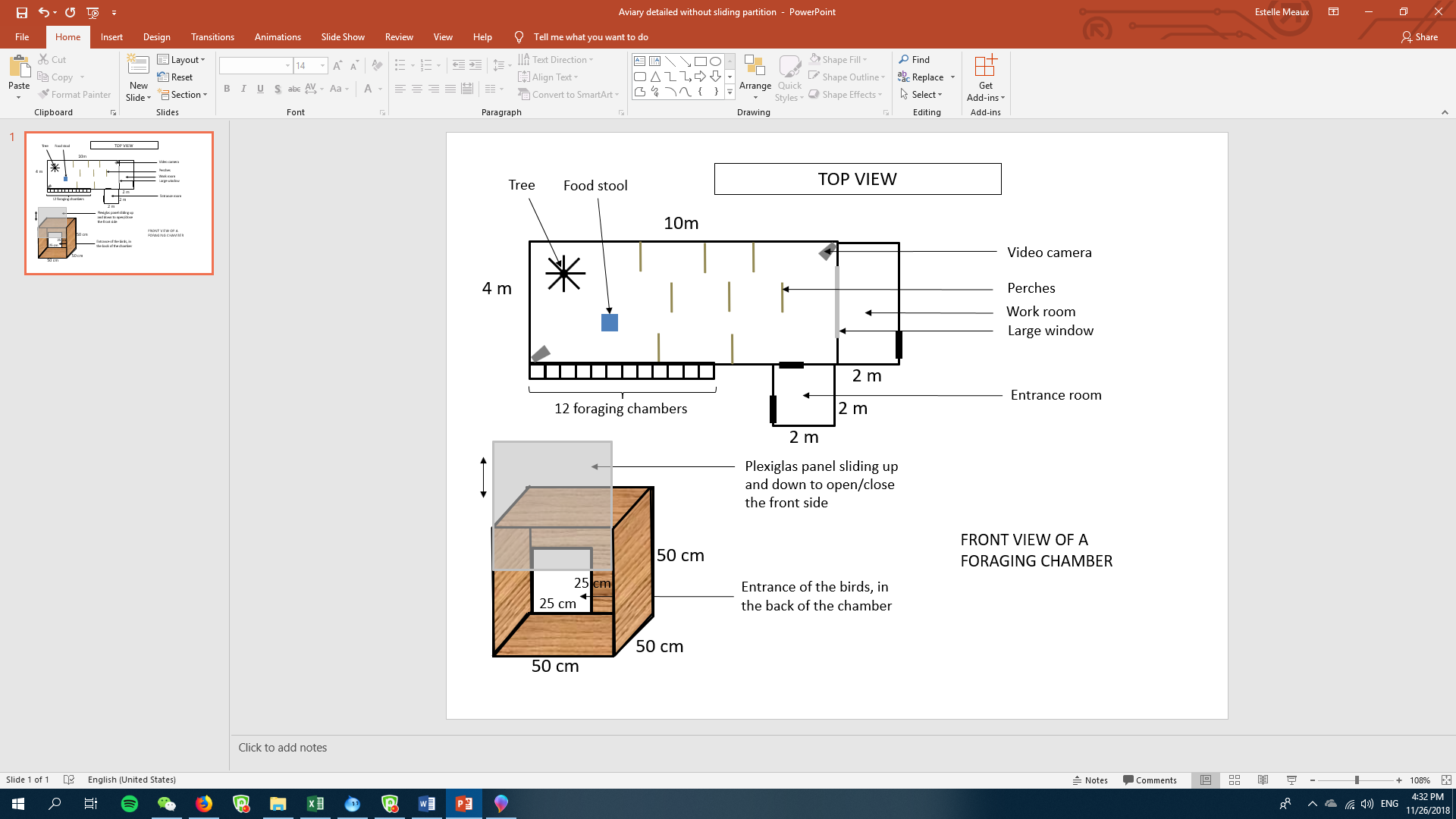
**

Figure S3. Predicted values of the rate of short-distance and flight calls for the subgroup sizes 1, 2 and 4 birds

, that is, using only data from the experimental stage and excluding the group size of 8 birds during the pre-experimental stage. All models are negative binomial GLMMs (see Table S2). The long-distance call is not shown because the variable of group size was not significant for it. *N* = 411 recordings.


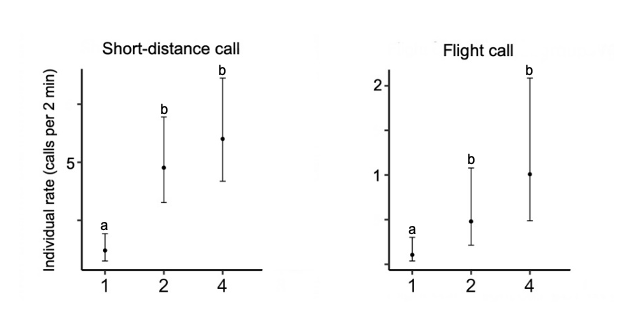

Supplement: Supplementary file 1 — Appendix S1 [file ECE3-13-e9909-s001.docx]
